# Pathophysiology of Prediabetes Hyperinsulinemia and Insulin Resistance in the Cardiovascular System

**DOI:** 10.3390/biomedicines13081842

**Published:** 2025-07-29

**Authors:** Ghassan Bkaily, Ashley Jazzar, Amira Abou-Aichi, Danielle Jacques

**Affiliations:** Department of Immunology and Cell Biology, Faculty of Medicine and Health Sciences, University of Sherbrooke, Sherbrooke, QC J1H 5N4, Canada; ashley.jazzar@usherbrooke.ca (A.J.); amira.abou-aichi@usherbrooke.ca (A.A.-A.)

**Keywords:** insulin, insulin receptor, insulin signaling, hyperinsulinemia, insulin resistance, type 2 diabetes, cardiovascular disease, NHE, NCX, ROS, obesity, calcium, excitation-secretion coupling, internalisation, prediabetes

## Abstract

Hyperinsulinemia refers to an elevated level of circulating insulin (80 and 100 µU/mL), often leading to metabolic disorders such as obesity, insulin resistance, and type 2 diabetes (T2D). There is no precise and universally accepted definition of hyperinsulinemia and insulin resistance. The literature in the field remains unclear regarding whether insulin resistance precedes the development of hyperinsulinemia. Recently, a new hypothesis has been proposed suggesting that chronic hyperinsulinemia precedes and causes insulin resistance. The causes of the initiation of hyperinsulinemia, insulin resistance, and type 2 diabetes are multifactorial. Thus, it is not easy to define in general. Recent work demonstrates that the main prediabetic factor leading to insulin resistance is chronic hyperinsulinemia. However, recent work in the literature proposes that relatively long-term hyperinsulinemia does precede insulin resistance and already promotes cardiovascular remodeling. This later may lead to the development of vascular diseases such as hypertension. Thus, defining hyperinsulinemia and insulin resistance, as well as their signaling pathways implicated in the development of type 2 diabetes (T2D), needs to be clarified.

## 1. Introduction

Prediabetes is defined as an intermediate metabolic state of dysglycemia characterized by elevated blood glucose levels that are not high enough to be categorized and diagnosed as diabetes [1]. It is a highly prevalent condition, particularly among older and obese individuals [1]. It is reported to be associated with insulin resistance and early β-cell dysfunction [2]. The multiple definitions that exist are based on differing thresholds of hemoglobin A1c (HbA1c, 5.7–6.4%), fasting glucose (100–125 mg/dL), and 2 h plasma glucose following a 75-g oral glucose tolerance test (OGTT) (140–199 mg/dL) [1]. Prediabetes constitutes a high risk for progression to type 2 diabetes, as well as an increased risk of developing several diseases such as cardiovascular disease, nephropathy, neuropathy, retinopathy, non-alcoholic fatty liver disease, and all-cause mortality [2,3]. Multiple metabolic, demographic, and lifestyle-related risk factors are associated with prediabetes, such as being overweight. Obesity is a chronic, multifactorial disease characterized by excessive accumulation of body fat that presents a health risk [4]. It is commonly classified based on Body Mass Index (BMI): overweight (BMI 25.0–29.9 kg/m^2^), class I obesity (30.0–34.9 kg/m^2^), class II obesity (35.0–39.9 kg/m^2^), and class III or severe/morbid obesity (≥40.0 kg/m^2^) [4]. A key metabolic consequence of obesity is hyperinsulinemia [5]. Adipose tissue in T2D stimulates the production of several cytokines, including TNF-alpha and IL-6 [6]. The etiology of obesity differs between the adult and pediatric populations [7].

The dogma views hyperinsulinemia as a compensatory response to insulin resistance in insulin-sensitive tissues such as muscle, liver, and adipose tissue [5]. However, recently, it has been suggested that hyperinsulinemia can also occur independently of diabetes as well as in congenital hyperinsulinism [8]. Thus, insulin resistance is a compensatory response to hyperinsulinemia [9,10]. Misfolding of proinsulin in the endoplasmic reticulum contributes to β-cell dysfunction, potentially impairing the quality of insulin secretion and promoting the development of hyperinsulinemia, which in turn leads to insulin resistance [11]. Furthermore, mutations in the insulin receptor can impair insulin signaling, leading to reduced glucose uptake and compensatory hyperinsulinemia, which ultimately contributes to the development of insulin resistance [12]. This bidirectional relationship highlights the complexity of metabolic disorder development, where hyperinsulinemia may initiate or exacerbate insulin resistance, particularly when insulin receptors are dysfunctional or when the secreted insulin exhibits reduced biological activity [11,12]. This elevated insulin output enhances glucose uptake in skeletal muscle and adipose tissue, helping to prevent hyperglycemia in the early stage [13].

Visceral adipose tissue secretes pro-inflammatory cytokines (TNF-α, IL-6) and free fatty acids [14]. This impairs insulin signaling and increases hepatic glucose production, perpetuating a cycle of hyperinsulinemia and insulin resistance [14]. Persistent hyperinsulinemia, exacerbates fat accumulation and further metabolic dysfunction, creating a self-reinforcing loop that increases the risk for T2D and cardiovascular disease [13]. Thus, as insulin resistance, hyperinsulinemia can be considered a prediabetes factor.

## 2. Hyperinsulinemia, Insulin Resistance, and Type 2 Diabetes

Produced by beta-pancreatic cells, insulin is one of the most critical circulating hormones that regulate cell metabolism by promoting glucose transport through the plasma membrane of all cells [15,16]. Its primary function is to maintain normal blood glucose levels between 15 and 45 µU/mL [17,18], whereas in hyperinsulinemia, insulin resistance, and type 2 diabetes, it ranges from 80 to 100 µU/mL [15,17,19,20]. Its circulating concentration depends on the level of glycemia [21]. The major glucose transporters in the plasma membrane of β-cells and in the cardiovascular system are GLUT2 and 4 [22]. The generation of ATP following glycolysis inhibits the ATP-dependent potassium channel (K_ATP_). This latter induces depolarization of the β-cell sarcolemma membrane to the level of the threshold of voltage-dependent calcium channels (VDCCs, mainly the L-type and R-type), leading to their opening and entry of calcium into the cell [23]. The increase in cytosolic calcium of β-cells promotes the fusion of insulin secretory vesicles with the plasma membrane, facilitating exocytosis and the release of the hormone into the circulation [18,22].

**Figure 1:**
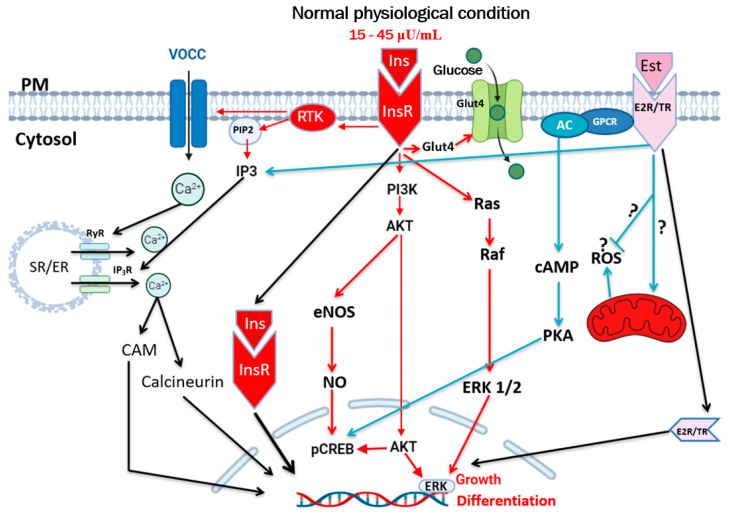
Schematic representation of main insulin receptor signaling and membrane transporters activated by normal levels of insulin (15–45 µU/mL). Ins, insulin; InsR, insulin receptor; VOCCs, voltage-operated calcium channel; Glut4, glucose transporter type 4; Est, estrogen; E2R/TR, estrogen receptor (E2), thyroid hormone receptor; SR/ER, endoplasmic reticulum; RyR, ryanodine receptor; IP_3_R, inositol 3 phosphate receptor; GPCR, G-protein coupled receptor; RTK, tyrosine kinase receptor; PM, plasma membrane; CaM, calmodulin; AC, adenylyl cyclase; cAMP, cyclic adenosine monophasphate; PKA, protein kinase A; Ras, rat sarcoma; Raf, rapidly accelerated fibrosarcoma; PI3K, phosphoionositide 3-kinase; AKT, protein kinase B; ERK, extracellular signal-regulated kinase; eNOS, endothelial nitric oxide synthase; NO, nitric oxide; pCREB, phosphorylated cAMP response element-binding protein; E2R/TR, estrogen receptor (E2), thyroid hormone receptor; Ca^2+^ represents the increase in calcium. The question mark (?) indicates that the effect on the mitochondria is a matter of debate.

Blood circulating insulin binds to its tyrosine kinase family (RTK) receptors, alpha and beta subunits of the plasma membrane of all cell types [18,24,25]. Activation of the beta subunit phosphorylates the insulin receptor substrate-1 (IRS-1), resulting in the generation of phosphatidylinositol 3,4,5-trisphosphate (PIP3) and the activation of AKT. The latter will promote translocation of the glucose transporter GLUT-4 to the plasma membrane [18] (Figure 1). For more information, please consult references from [9,17,18,20,26].

Genetic, environmental, and dietary factors contribute to the overproduction of insulin by beta cells [27]. The sustained increase in circulating insulin is known as hyperinsulinemia [27]. It is considered a defense mechanism of beta cells against persistent hyperglycemia [28]. Hyperinsulinemia can occur in the presence or absence of hyperglycemia, such as during in-utero development, postnatal development, and adolescence [7,9,29]. There is no precise and universally accepted definition of hyperinsulinemia [20,27,30]. Hyperinsulinemia is asymptomatic or poorly symptomatic; therefore, it takes several years before leading to diabetes [17]. Individuals with hyperinsulinemia may not necessarily meet any diagnostic criteria for metabolic syndrome [7]. It is considered high risk for cardiovascular complications combined with obesity, diabetes, and hypertension [7]. It can be caused during the pediatric age by molecular alterations in insulin secretion or in insulin receptor responses [7].

Although it is well-accepted that insulin resistance precedes type 2 diabetes mellitus [9,18], the literature in the field remains unclear regarding whether insulin resistance precedes the development of hyperinsulinemia [9,18,19]. Recently, a new hypothesis has been proposed suggesting that chronic hyperinsulinemia precedes and causes insulin resistance [9].

Furthermore, it is also proposed that insulin resistance may serve as a mechanism of defense against chronic hyperinsulinemia, thereby preventing the over-signaling of insulin receptors [13,16,18] that leads to the remodeling of various cell types, including those of the cardiovascular system [20,31]. We must mention that the causes of the initiation of hyperinsulinemia, insulin resistance, and type 2 diabetes are multifactorial. Thus, it is not easy to define it in general.

**Figure 2:**
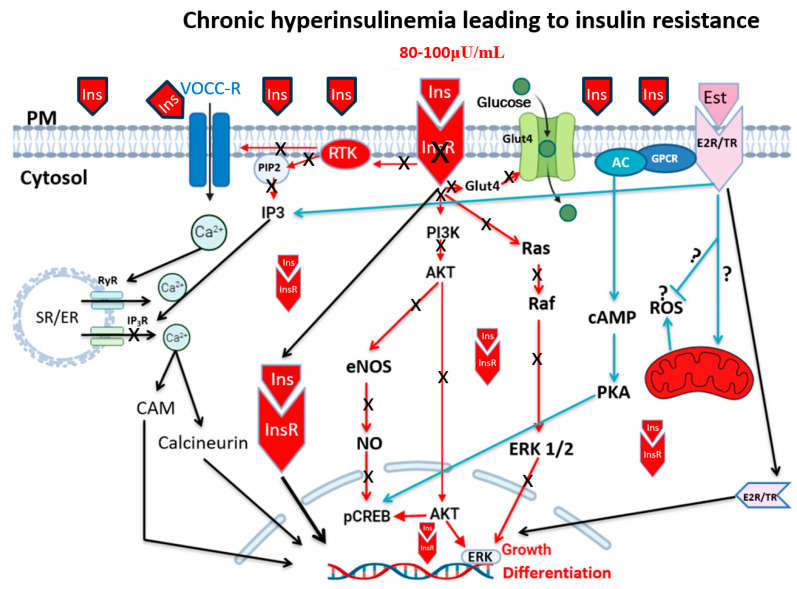
Schematic representation of the effect of hyperinsulinemia (80–100 μU/mL)-induced insulin resistance. Overstimulation of insulin receptors by hyperinsulinemia-induced internalization of insulin receptor complex as well as its translocation into the nucleus. The decrease in density of plasma membrane insulin receptors shuts down (indicated by X). However, the internalized and translocated complex of insulin and its receptors continues intracellular signaling. Activation of voltage-dependent R-type calcium channel (VOCC-R) increases intracellular calcium, which in turn releases calcium via the ryanodine receptor (RyR) by calcium-induced calcium release mechanism. The continuous activation of pCREB will prevent a change in the phenotype of VSMCs. Ins, insulin; InsR, insulin receptor; VOCCs, voltage-operated calcium channel; Glut4, glucose transporter type 4; Est, estrogen; E2R/TR, estrogen receptor (E2), thyroid hormone receptor; SR/ER, endoplasmic reticulum; RyR, ryanodine receptor; IP_3_R, inositol 3 phosphate receptor; GPCR, G-protein coupled receptor; RTK, tyrosine kinase receptor; PM, plasma membrane; CaM, calmodulin; AC, adenylyl cyclase; cAMP, cyclic adenosine monophasphate; PKA, protein kinase A; Ras, rat sarcoma; Raf, rapidly accelerated fibrosarcoma; PI3K, phosphoionositide 3-kinase; AKT, protein kinase B; ERK, extracellular signal-regulated kinase; eNOS, endothelial nitric oxide synthase; NO, nitric oxide; pCREB, phosphorylated cAMP response element-binding protein; E2R/TR, estrogen receptor (E2), thyroid hormone receptor; Ca^2+^ represents the increase in calcium. The question mark (?) indicates that the effect on the mitochondria is a matter of debate.

When comparing hyperinsulinemia and insulin resistance, it is also important to consider the duration of hyperinsulinemia, which can be categorized as acute, subacute, or chronic. It is logical to conclude that the short-term increase in hyperinsulinemia will not necessarily promote the development of insulin resistance; however, both acute and prolonged exposure may have this effect. It is accepted that among the initiators or causes of hyperinsulinemia are high calorie intake and specific types of diets, such as those exceeding 5000 kcal/day. Hyperinsulinemia increases insulin receptor signaling and rapid internalization, thereby decreasing the density of plasma membrane receptors (Figure 2). This latter aspect promotes the development of insulin resistance and a subsequent decrease in receptor signaling, leading to increased glucose accumulation, which in turn supports further insulin secretion. This condition worsens chronic hyperinsulinemia and the development of type 2 diabetes. Although the dogma suggests that insulin resistance precedes the development of hyperinsulinemia, we and others support a new concept in which acute–chronic conditions can also precede the development of insulin resistance, ultimately leading to type 2 diabetes [17,18,20,26] (Figure 2). It is also believed that chronic hyperinsulinemia is secondary to insulin resistance, which is considered a silent killer [17].

A diet high in calories will also lead to obesity [15,20], which may promote the development of type 2 diabetes. However, this aspect is still a matter of debate since individuals can be obese without developing type 2 diabetes [32]. Therefore, early postnatal overnutrition may contribute to chronic effects on insulin secretion [9,33].

Hyperinsulinemia can take place with and without hyperglycemia [26,34,35]. Glucose is the primary energy source for cells. When elevated, it induces insulin secretion into the circulation, allowing its uptake into the cell via GlUT2 and 4 (Figure 2). One crucial aspect of the increase in circulating glucose (hyperglycemia) is that it increases extracellular osmolarity, which obliges the cells to compensate, leading to abnormal intracellular homeostasis. Chronic elevation of circulating insulin during chronic hyperinsulinemia activates its receptors (Figure 1), thus promoting high hyperglycemia toward lipogenesis. This process supports fat storage while inhibiting lipolysis, leading to high fat accumulation and obesity [36,37]. This latter aspect is supported by a clinical study showing that pharmaceutical reduction in insulin secretion leads to a decrease in body weight in obese individuals [38]. Thus, chronic overnutrition leads to hyperinsulinemia, and if sustained over time, it will contribute to the development of obesity [9]. Hyperinsulinemia is not only implicated in type 2 diabetes and obesity but also has a role in the pathology of the cardiovascular system. There is evidence that glucocorticoids, antipsychotic medications, cardiovascular medications (including statins, beta blockers, and diuretics), certain anti-infectives, antineoplastic medications, immunosuppressive agents, and hormonal treatments are associated with changes in glucose metabolism and an increased incidence of hyperglycemia and/or diabetes [39].

## 3. Hyperinsulinemia and Vascular Diseases

The cardiovascular system is one of the first systems to develop during embryogenesis and is the most extensive organ system in vertebrates [40,41]. It is a vast network of interconnected vessels that supply and drain blood to tissues, facilitating the elimination of metabolic byproducts and ensuring the body’s proper functioning [42,43]. It mainly consists of the venous and arterial systems [44].

The venous system is an afferent vessel operating in a low-pressure system, such as veins and venules [44]. The arterial system is an efferent vessel operating in a high-pressure system [44]. Hyperinsulinemia will first affect the vascular endothelium and promote its remodeling, which affects blood vessel function, leading to vascular diseases such as hypertension and atherosclerosis.

## 4. Hyperinsulinemia, Hypertension, and Atherosclerosis

Essential arterial hypertension (EAH) is the most prevalent form of hypertension, accounting for approximately 90% of all cases [45,46]. EAH is commonly classified into two categories [45]: 1 and 2 [45]. The precise mechanisms underlying the development of EAH remain difficult to identify [47]. However, recent epidemiological studies suggest that factors such as gender, age, lifestyle, and sex may play a role in the development of EAH [48].

Sustained elevated circulating insulin levels can have a direct impact on blood vessel morphology and function, thus contributing to the pathophysiology of the cardiovascular system, including secondary arterial hypertension (SAH) [44]. The hyperinsulinemia-induced remodeling of the heart and blood vessels leads to increased peripheral resistance and higher blood pressure [20,31,49]. Hyperinsulinemia plays a crucial role in the development of hypertension [50]. Furthermore, insulin resistance is identified as a key element in the development of hypertension [51]. This is attributed to a disruption in insulin signaling, which disrupts vascular homeostasis (Figure 2), leading to a cascade of pro-hypertensive mechanisms [50]. At the vascular level, insulin stimulates nitric oxide (NO) production via the PI3K-Akt-eNOS pathway (Figure 2), thereby facilitating vessel dilation [49,52]. However, in insulin resistance, the hormone primarily promotes the activation of the MAPK pathway (Figure 2), thereby intensifying vasoconstriction, vascular smooth muscle cell proliferation, inflammation, and the formation of atherosclerotic plaques. This disruption of signaling pathways is at the root of atherosclerosis, endothelial dysfunction, and vascular stiffness, both of which are hallmarks of hypertension [49,52]. Indeed, hyperinsulinemia also activates the renin-angiotensin-aldosterone system, promoting vasoconstriction and sodium retention [53]. These additive effects set up a cycle of worsening vascular lesions and hypertension [53].

**Figure 3:**
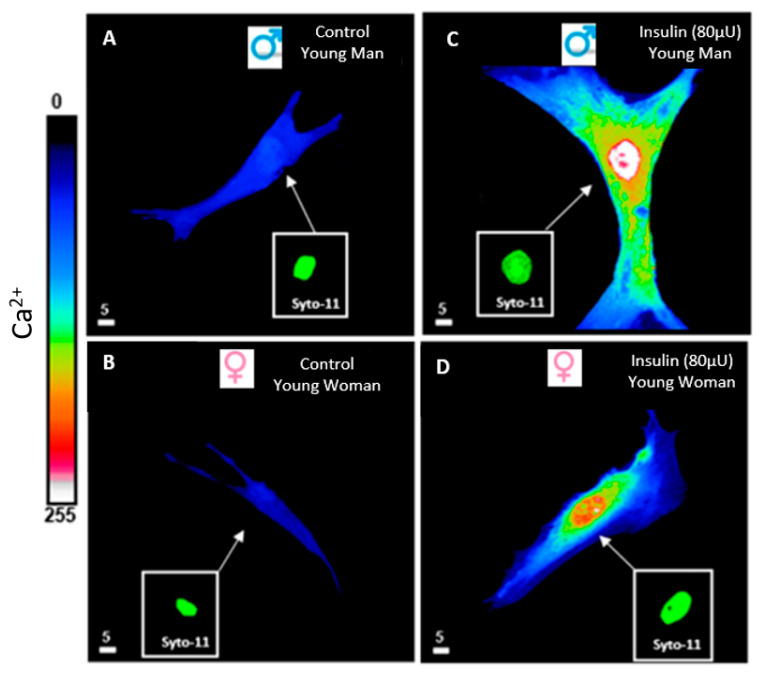
Effect of 48 h treatment of hyperinsulinemia (80 μU/mL) on intracellular Ca^2+^ levels of VSMCs from men and women. Examples of a typical quantitative 3D top-view image of VSMCs: in the absence of hyperinsulinemia in VSMCs from men (**A**) and women (**B**), and with hyperinsulinemia in VSMCs from men (**C**) and women (**D**). Panels (**C**,**D**) show an increase in intracellular Ca^2+^ levels of VSMCs from both men and women induced by 48 h of hyperinsulinemia. Inset panels (green staining) represent nuclear labeling of the cells observed in panels (**A**–**D**) with Syto-11. The pseudocolor scale represents Ca^2+^ fluorescence intensity ranging from 0 (no fluorescence) to 255 (maximum fluorescence). The white scale bar is μm. Cont: control, Ins: insulin. Modified from [20].

**Figure 4:**
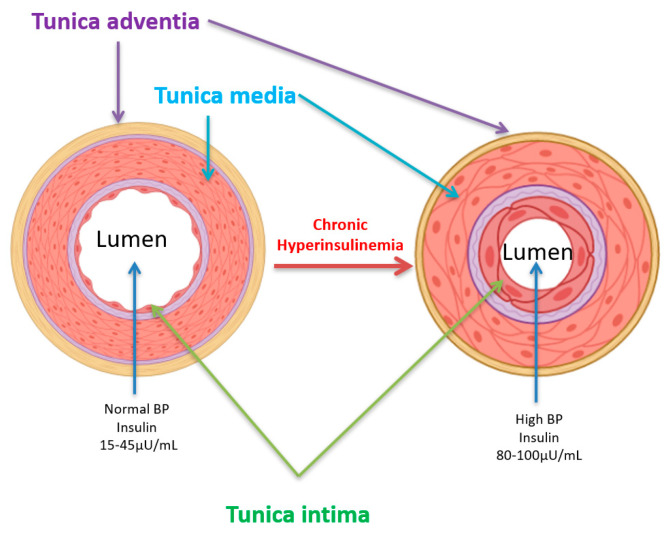
Schematic diagram of hyperinsulinemia hypertrophic remodeling characterized by a thickening of the vascular wall.

Furthermore, epidemiological meta-analyses and mechanistic research have revealed an increase in the plasma catecholamines system in association with hyperinsulinemia in hypertensive individuals who frequently exhibit hyperinsulinemia or insulin resistance [54]. Our recent work demonstrated that prolonged hyperinsulinemia for 48 h led to hypertrophy of cardiomyocytes and vascular smooth muscle cells, as well as elevated intracellular calcium (Ca^2+^) [6,18,31] (Figure 3), both of which are known to contribute to vascular dysfunction and hypertension. The hypertrophy of vascular endothelial and smooth muscle cells leads to the thickening of the tunica intima and media, resulting in a decrease in lumen volume and an increase in blood pressure (Figure 4). Vascular hypertrophy has been observed in diabetic rat models [50]. Consequently, hyperinsulinemia-related hypertension contributes to damage to target organs, such as the heart, with left ventricular hypertrophy, kidney disease, and carotid atherosclerosis [55]. Hyperinsulinemia and insulin resistance can have a direct impact on vessel function, participating in the pathophysiology of the cardiovascular system, including secondary arterial hypertension (SAH) [44]. The process involves insulin-induced remodeling of the heart and blood vessels, resulting in increased peripheral resistance and elevated blood pressure [49] (Figure 4). The deregulation of signaling pathways, particularly in the tunica intima, by hyperinsulinemia may lead to the development of atherosclerosis. This disruption of signaling pathways promotes endothelial dysfunction and vascular stiffness, both of which are hallmarks of hypertension [49,52].

## 5. Hyperinsulinemia and Calcium Ionic Transporters

Plasma membrane ionic calcium transporters that regulate directly or indirectly beta cells’ intracellular calcium homeostasis are voltage-operated calcium channels (VOCCs), receptor-operated calcium channels (ROCCs) [23,56], sodium-calcium exchangers (NCX) [57], sodium-hydrogen exchangers (NHE) [58,59,60], and the calcium ATPase [61,62]. Reactive oxygen species (ROS) also indirectly regulate intracellular calcium homeostasis by modulating calcium transporters [23].

In normal physiological conditions, VOCCs are selective to extracellular calcium and are key in regulating various cellular functions, including excitation-secretion and excitation-contraction coupling [23,63]. In the cardiovascular system as well as beta cells, three types of voltage-gated calcium channels (VOCCs) are present: T-, L-, and R-type calcium channels [64,65,66]. In the vascular endothelium, only the R-type is present [7], whereas in vascular smooth muscle cells, both the L- and R-type VOCCs are present [67,68]. Since the resting potential of VSMCs is near −65 mV, the L-type is responsible for calcium influx during short depolarization, and the R-type is accountable for maintaining tension during maintained depolarization [23,69].

The steady-state R-type calcium channel was reported to be activated by insulin [69,70]. L-type channels are not present in early cardiovascular development.

They appeared during cardiovascular differentiation. Insulin does not stimulate this type of channel [71]. The R-type calcium channel, or the resting Ca^2+^ channel, regulates resting calcium homeostasis [23,69]. This steady-state calcium channel is the only Ca^2+^ channel present at the nuclear level of excitable and non-excitable cells, contributing to nuclear Ca^2+^ regulation [23,69]. Hyperinsulinemia stimulates and activates R-type calcium channels, leading to calcium overload and activation of a signaling pathway implicated in the development of hypertrophy [69,70]. Recent work from our laboratory showed that hyperinsulinemia induced sustained cytosolic and nuclear calcium overload in human VSMCs from men and women [20]. Thus, this type of channel can be a good target for preventing hyperinsulinemia-induced cardiovascular remodeling.

Another essential calcium transporter is the NCX [72]. Hyperinsulinemia, as well as insulin resistance, regulates glucose transporter-mediated inorganic phosphate, which is implicated in the formation of ATP, cyclic AMP, and the phosphorylation of proteins, including Na/phosphate exchanger [71] and NCX [72]. Both exchangers are stimulated by insulin [72,73]. This exchanger plays an important role in physiology and pathology, as well as in early cardiovascular embryonic development, by regulating calcium influx [23]. NCX1 expression has been reported to be upregulated in animal models of cardiac hypertrophy [74]. However, whether NCX1 is at least partially responsible for hyperinsulinemia-induced VSMC remodeling remains to be elucidated.

Another type of exchanger that regulates intracellular calcium homeostasis indirectly via intracellular sodium is the sodium-hydrogen exchanger [58]. The main NHE isoforms implicated in insulin action and secretion are NHE1, NHE2, NHE3 [75], and NHA2 [76]. Isoforms NHE-1 to NHE-5 are located on the plasma membrane of various cell types, while isoforms NHE-6 to NHE-10 are found on intracellular membranes [58]. These isoforms are expressed differently across tissues, with NHE-1 being the most prominent and ubiquitously expressed in the cytosolic and nuclear membranes of various cell types, including VSMCs [77,78,79]. Insulin has been reported to increase the activity [80] and expression [81] of NHE via insulin signaling. Glucagon-like peptide-1 receptor agonists and the sodium-glucose cotransporter-2 inhibitors (SGLT2i) prevent endothelial dysfunction by regulating the sodium-hydrogen exchangers, NHE1 and NHE3 [82]. As the NCX, the role of NHE in hyperinsulinemia and insulin resistance needs to be explored.

## 6. Hyperinsulinemia and Reactive Oxygen Species (ROS)

A key factor in diabetes is oxidative stress [6]. It is well-established that oxidative stress is associated with type 2 diabetes [83]. Although the precise mechanism of how type 2 diabetes induces oxidative stress remains unclear, it may stem from mitochondrial dysfunction and increased ROS production via NOX proteins [20,84]. A sustained increase in ROS is the hallmark of oxidative stress in T2D [6,85]. This intracellular accumulation of ROS occurs when the capacity of the antioxidant system is unable to chelate the accumulation of ROS [86]. The excessive accumulation of ROS induces various types of damage to cells, including lipid membranes, proteins, DNA, and insulin production in beta cells; these damages promote inflammation [6,85]. ROS can initiate and amplify an inflammatory cascade, and this later activation activates immune cells, promoting further ROS generation [6]. Antioxidant levels are lower in obese individuals compared to those of normal weight [6]. The relationship between antioxidants and individuals with diabetes can be, at least in part, due to differences in genetic background [6]. Studies in type 2 diabetic mouse models have shown that mitochondrial dysfunction and ROS generation are significantly higher compared to those in normal mice [87] and humans [85]. Furthermore, it has been demonstrated in vitro that hyperinsulinemia increases vascular ROS levels [20,88] (Figure 1 and Figure 2). This was shown in a study using isolated arterioles from skeletal muscle and VSMCs from human aorta, which showed that treatment with hyperinsulinemia increases ROS levels [20,88] (Figure 1 and Figure 2). Increased ROS levels lead to elevated intracellular Ca^2+^ by inducing stress on the endoplasmic reticulum (ER), which activates calcium channels [89]. The ER serves as a key storage site for Ca^2+^, with Ca^2+^ released primarily through insulin-induced activation of inositol 1,4,5-trisphosphate receptors (IP_3_R) and ryanodine receptors (RyR) [20,90] that insulin activates. Excessive ROS can stress the ER and modulate RyR activity, triggering the release of Ca^2+^ [89]. This rise in intracellular Ca^2+^ activates CREB, calmodulin, and calcineurin, which then dephosphorylate NFAT, promoting morphological remodeling [91] (Figure 1 and Figure 2). It has been demonstrated that CREB is the guardian of the contractile VSMCs phenotype, able to regulate antioxidant gene expression directly [44,92]. Furthermore, it was shown that hyperinsulinemia decreases CREB in cardiomyocytes [31] (Figure 2). Although the impact of hyperinsulinemia on the vascular system, particularly on VSMCs, is still not fully understood, most studies on hyperinsulinemia, insulin resistance, and type 2 diabetes have primarily focused on males. However, recent epidemiological research has shown that hypertension and type 2 diabetes are influenced by various factors, including sex [20,93].

**Figure 5:**
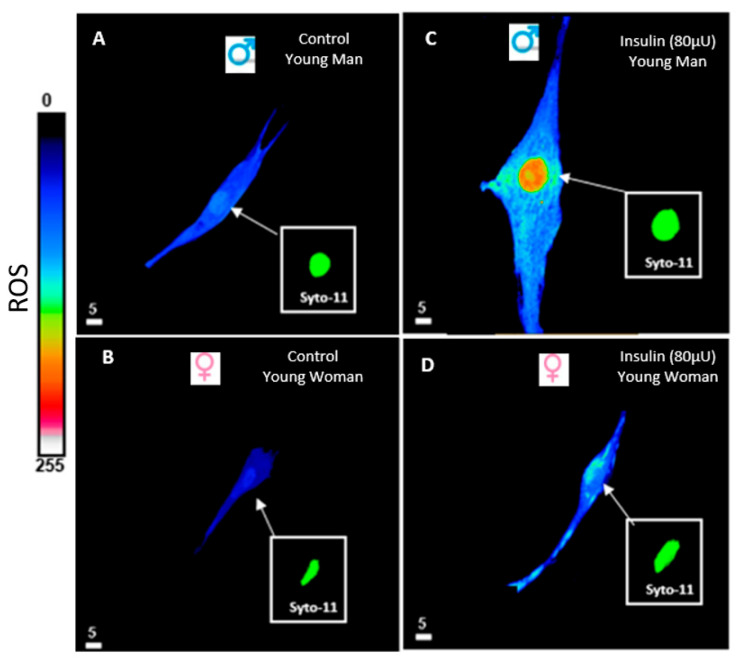
Effect of 48-h insulin treatment (80 μU) on the intracellular ROS levels of VSMCs in Men and Women. Examples of a typical quantitative 3D top-view image of VSMCs under normal conditions in men (**A**) and women (**B**), and with a hyperinsulinemia treatment in men (**C**) and women (**D**). Panels C and D show a noticeable increase in intracellular ROS levels of VSMCs in both men (**C**) and women (**D**) induced by 48 h of hyperinsulinemia. However, the cellular and nuclear ROS levels of VSMCs in men treated with hyperinsulinemia appear larger than those of the women treated under the same conditions. The inset panels (green staining) represent the nuclear labeling of the cells observed in panels (**A**–**D**) with Syto-11. The pseudocolor scale represents ROS fluorescence intensity ranging from 0 (no fluorescence) to 255 (maximum fluorescence). The white scale is measured in μm. Modified from [20].

Evidence suggests that reactive oxygen species (ROS) play a key role in the development of insulin resistance. These are generated by mitochondria [94] (Figure 1). An imbalance of ROS and anti-ROS leads to a disruption of redox homeostasis and promotes insulin resistance [94,95]. Recent work from our group has shown that hyperinsulinemia induces a significant increase in ROS and a decrease in glutathione in VSMCs in both men and women [20] (Figure 5).

Overall, clinical trials exploring sex differences and potential therapeutic strategies for hyperinsulinemia and insulin resistance remain limited. Moreover, while sex differences between men and women have been observed, studying the direct effects of hyperinsulinemia on VSMCs or intima-media thickness in patients is not feasible in a clinical setting. Therefore, in vitro studies provide a valuable approach to investigating these aspects, including the impact of hyperinsulinemia on vascular remodeling and secondary hypertension [20].

Most studies on hyperinsulinemia have been conducted using animals [31,88]. While these models provide valuable insights, they do not fully replicate human physiology. Additionally, despite evidence of sex differences in hyperinsulinemia, they are often overlooked in research.

## 7. Discussion, Conclusions, and Perspectives

The dogma taught us that insulin resistance is the main prediabetic pathological condition that leads to obesity and diabetes type-2 [9,18,26]. However, recent work demonstrates that the main prediabetic factor leading to insulin resistance (desensitization of insulin receptor) is chronic hyperinsulinemia [18,20]. In addition, the dogma teaches us that insulin resistance is caused by the oversecretion of insulin by beta cells, resulting from genetic disorders and overnutrition, which leads to the development of obesity [9,96]. However, recent work in the literature proposes that relatively long-term hyperinsulinemia does precede insulin resistance and already promotes cardiovascular remodeling. In addition, chronic long-term hyperinsulinemia may lead to insulin resistance, in part by promoting the rapid internalization of the insulin/receptor complex, which reduces the density of cell membrane insulin receptors. This results in a decrease in insulin receptor signaling and the uptake of extracellular insulin, as well as a reduction in the expression of glucose transporters. All these factors would lead to a decrease in insulin action, which is distinct from a loss or decrease in insulin sensitivity. Thus, it is extremely important to understand the reasons leading to hyperinsulinemia and its pathophysiological actions, which result in desensitization of excitation-contraction and excitation-secretion coupling. Finally, regarding glucose, routine measurement of circulating insulin levels would certainly help detect the development of hyperinsulinemia, thereby preventing insulin resistance and the progression to T2D. In addition, hyperinsulinemia as well as ROS levels are good markers for the detection of prediabetes in the absence of asymptomatic or poorly symptomatic silent killer.

## Data Availability

Data are contained within the article.
